# Supplementary material for: Preparatory Conditions Optimization and Characterization of Hierarchical Porous Carbon from Seaweed as Carbon-Precursor Using a Box—Behnken Design for Application of Supercapacitor
Source: Materials (Basel). 2022 Aug 20;15(16):5748. doi: 10.3390/ma15165748 (PMC9416258; doi:10.3390/ma15165748)
Supplement: Supplementary file 1 [file materials-15-05748-s001.zip › materials-1833372-supplementary.pdf]

Supplementary ,material

Preparatory Conditions Optimization and Characterization of Hierarchical Porous Carbon from Seaweed as Carbon-Precursor Using a Box—Behnken Design for Application of Supercapacitor

Wein-Duo Yang [1,*], Jing-Xuan Wang [1], Yu-Tse Wu [2], Hsun-Shuo Chang [2] and Horng-Huey Ko [2]

[1] Department of Chemical and Materials Engineering, National Kaohsiung University of Science and
Technology, Kaohsiung City 807, Taiwan

[2] School of Pharmacy, College of Pharmacy, Kaohsiung Medical University, Kaohsiung City 807, Taiwan

* Correspondence: ywd@nkust.edu.tw

Table S1. A part of the DOE experimental result.

Std. No.	Run	A Temperature (℃)	B Time (min)	C NaCl/SW (weight ratio)	D Water/SW (weight ratio)	Response Specific Capacitance (F/g)	σ of C_s (F/g)
25	1	700	60	3	10	99.8 (±1.4)	3.1
19	2	600	60	5	10	86.3 (±1.9)	4.3
22	3	700	90	4	7.5	87.9 (±1.4)	3.1
9	4	600	60	4	7.5	76.9 (±1.7)	3.8
4	5	800	90	4	10	54.1 (±0.7)	1.6
6	25	700	60	5	7.5	69.7 (±2.3)	5.1

For example, the run 1 of electrode prepared from the conditions of A, B, C, and D, the specific capacitance (C_s) was measured 5 times by GCD test. The specific capacitance of run 1 electrode was measured at 96.6 F g^{-1}, 97.7 F g^{-1}, 98.4 F g^{-1}, 102.7 F g^{-1}, and 103.6 F g^{-1}, respectively. The data are calculated by statistical method as follows.

The experimental data is expressed as follows:

$$x = \bar{X} \pm \sigma_{\bar{x}} \qquad (1)$$

x: Results of multiple capacitance (here is C_s)

$\bar{X}$: mean of measured data

$\sigma_{\bar{x}}$: standard error of the mean

σ: standard deviation of the data

$\bar{X}$= (96.6 + 97.7 + 98.4 + 102.7 + 103.6) ÷ 5
= 99.8

For a limited number of specific capacitance, n= 5

$$\sigma = \sqrt{\frac{\sum d_i^2}{(n-1)}} \qquad (2)$$

$$\sigma_{\bar{x}} = \sqrt{\frac{\sum d_i^2}{n(n-1)}} \qquad (3)$$

$$d = x - \bar{x} \qquad (4)$$

$$\sigma = \sqrt{\frac{(96.6-99.8)^2+(97.7-99.8)^2+(98.4-99.8)^2+(102.7-99.8)^2+(103.6-99.8)^2}{(5-1)}} \qquad (5)$$

= 3.1

$$\sigma_{\bar{x}} = \sqrt{\frac{(96.6-99.8)^2+(97.7-99.8)^2+(98.4-99.8)^2+(102.7-99.8)^2+(103.6-99.8)^2}{5\times(5-1)}} \qquad (6)$$

= 1.4

$$Cs = x = \bar{X} \pm \sigma_{\bar{x}} \qquad (1)$$

= 99.8± 1.4

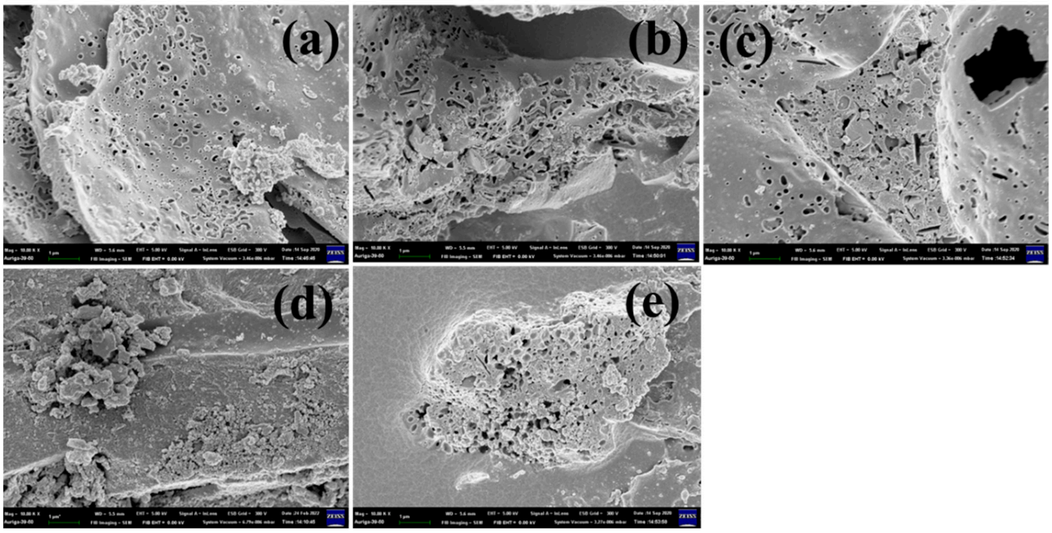

Figure S1. The surface morphology of activated carbon samples examined by SEM on magnification of 10,000 (a) run 15, (b) run 20, (c) run 21, (d) run 2, (e) optimal.

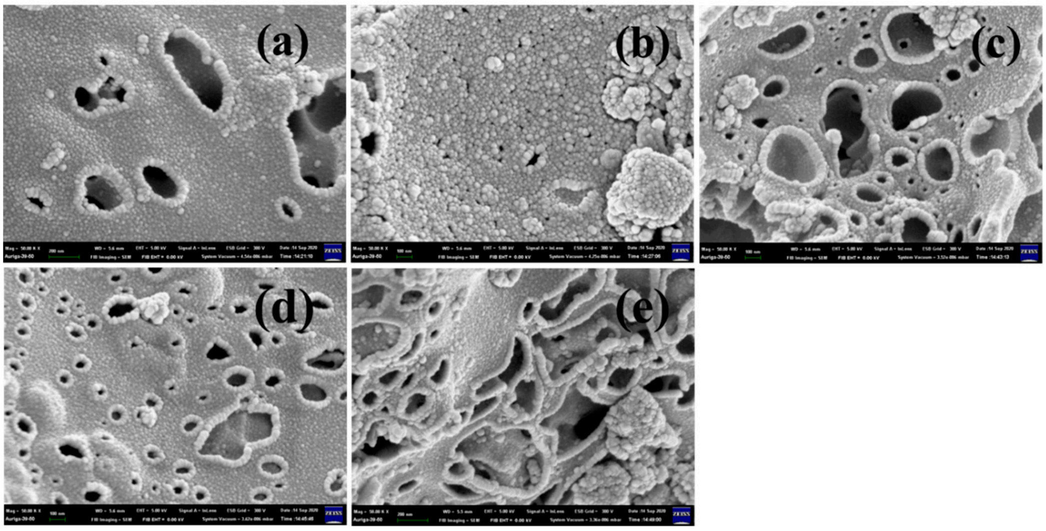

Figure S2. The surface morphology of activated carbon samples examined by SEM on magnification of 50,000 (a) run 15, (b) run 20, (c) run 21, (d) run 2, (e) optimal.

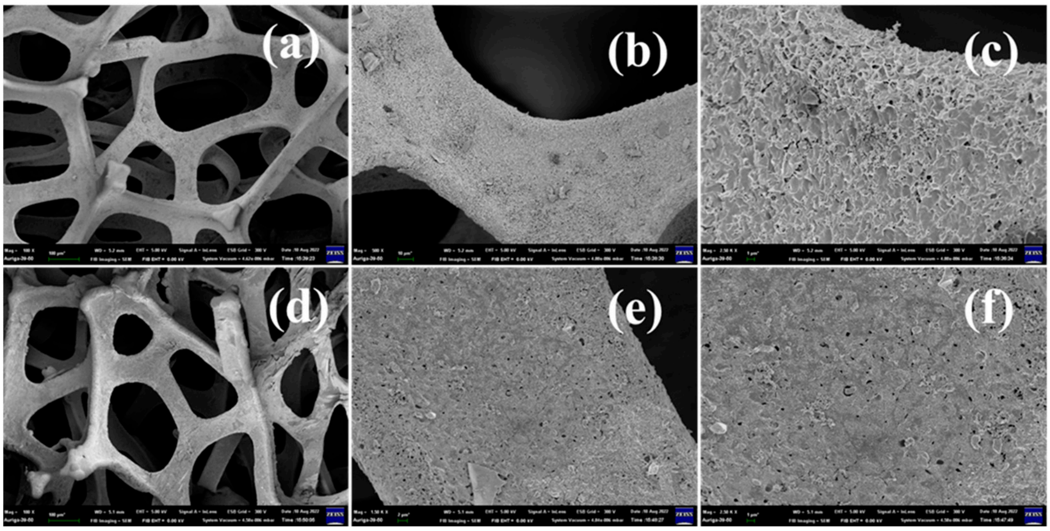

Figure S3. The optimal electrode examined by SEM before/after 10,000 charge/discharge cycles test. Before test on magnification at 150, 1500, and 2500, respectively(a, b, c); after test on magnification at 150, 1500, and 2500, respectively (d, e, f).
